# Noninvasive Vascular Displacement Estimation for Relative Elastic Modulus Reconstruction in Transversal Imaging Planes

**DOI:** 10.3390/s130303341

**Published:** 2013-03-11

**Authors:** Hendrik H.G. Hansen, Michael S. Richards, Marvin M. Doyley, Chris L. de Korte

**Affiliations:** 1 Medical UltraSound Imaging Center (MUSIC), Department of Radiology, Radboud University Nijmegen Medical Center, P.O. Box 9101, Nijmegen 6500 HB, The Netherlands; E-Mail: c.dekorte@rad.umcn.nl; 2 Department of Electrical and Computer Engineering, Hajim School of Engineering and Applied Sciences, University of Rochester, Hopeman Engineering Building, P.O. Box 270126, Rochester, NY 14627, USA; E-Mails: michael.richards@rochester.edu (M.S.R.); doyley@ece.rochester.edu (M.M.D.)

**Keywords:** strain imaging, modulography, beam steering, vascular ultrasound, vulnerable plaque, elastography, compounding

## Abstract

Atherosclerotic plaque rupture can initiate stroke or myocardial infarction. Lipid-rich plaques with thin fibrous caps have a higher risk to rupture than fibrotic plaques. Elastic moduli differ for lipid-rich and fibrous tissue and can be reconstructed using tissue displacements estimated from intravascular ultrasound radiofrequency (RF) data acquisitions. This study investigated if modulus reconstruction is possible for noninvasive RF acquisitions of vessels in transverse imaging planes using an iterative 2D cross-correlation based displacement estimation algorithm. Furthermore, since it is known that displacements can be improved by compounding of displacements estimated at various beam steering angles, we compared the performance of the modulus reconstruction with and without compounding. For the comparison, simulated and experimental RF data were generated of various vessel-mimicking phantoms. Reconstruction errors were less than 10%, which seems adequate for distinguishing lipid-rich from fibrous tissue. Compounding outperformed single-angle reconstruction: the interquartile range of the reconstructed moduli for the various homogeneous phantom layers was approximately two times smaller. Additionally, the estimated lateral displacements were a factor of 2–3 better matched to the displacements corresponding to the reconstructed modulus distribution. Thus, noninvasive elastic modulus reconstruction is possible for transverse vessel cross sections using this cross-correlation method and is more accurate with compounding.

## 1. Introduction

Rupture of atherosclerotic plaques and the successive formation of thrombus is regarded as one of the major causes of stroke and myocardial infarction [1–4]. The rupture proneness of a plaque is determined by its geometry and composition, and furthermore related to the amount of force that the pulsating blood exerts on the plaque [1,4–8]. It is known that 60 to 80% of strokes and myocardial infarctions are caused by the rupture of a plaque that has a large inflammatory lipid core which is covered by a thin fibrous cap that separates the lipid content from the blood in the lumen [1,3–5]. Because lipids have different elastic properties than fibrous and calcified tissue [9], it can be expected that vulnerable plaques deform differently in response to the pulsating blood. Ultrasound strain imaging aims at the measurement of these deformations and with that at the identification of high risk plaques. However, strain imaging does not provide information on the exact size and position of the lipid-rich core, because strain is an indirect measure of the elastic properties of the tissue. On the other hand, visualization of the elastic moduli inside the vessel wall would provide such information, because the elastic modulus is an intrinsic tissue property. Knowledge of the lipid-core size is very useful for instance for pharmaceutical trials in which the reduction of lipid content by administration of medication is aimed for.

Elastic modulus reconstructions have been performed for coronary arteries based on ultrasound strain information that was derived from raw radiofrequency (RF) data obtained intravascularly using a catheter-mounted ultrasound device [10–16]. However, in the last couple of years techniques for the noninvasive assessment of strains in the carotid artery wall and plaque have also been developed [17–26]. These noninvasive studies illustrate that accurate strain estimates can be obtained, despite the fact that ultrasound transmit frequencies are a factor of two to four lower than those applied in IVUS imaging. Of course the aim is no longer at strain estimation in the small and deeply located coronary arteries, but in the superficial and larger carotid artery. For a noninvasive assessment of carotid artery strains, the ultrasound data are usually acquired with a linear array transducer. In most studies the transducer is placed parallel to the direction of the vessel axis, because it allows a direct estimation of the radial strain in the vessel wall, since the ultrasound beam direction corresponds to the direction of the radial strain. Several publications on elastic modulus reconstruction for the carotid artery in this longitudinal imaging plane can be found [20,27]. At this moment no study exists in which modulus reconstructions are performed for entire transverse cross sections of carotid arteries given displacement estimates derived from ultrasound RF data acquired with a linear array transducer. In this study we do present relative elastic modulus reconstructions for vessel mimicking phantoms in that imaging plane. In order to perform reconstructions for this imaging plane, accurate estimates are required of both the displacements in the direction of the ultrasound beam (axial) and the displacements in the direction perpendicular to the ultrasound beam (lateral). From conventional single-angle acquisitions it is usually possible to obtain accurate estimates in the axial direction only. Displacements in the lateral direction are less accurate, due to the lack of phase information and the lower resolution in that direction. The lateral displacement estimates can be improved by compounding of displacement or strain estimates obtained at multiple acquisition angles [17,28–32]. The different acquisition angles are obtained by electronically changing the transmit delays of the adjacent piëzo-electric elements of a linear array transducer, called beam steering. Recently, we proposed a method for estimation of the full 2D displacement vector by projection of axial displacements estimated using ultrasound RF data obtained at three different acquisition angles [17]. The root mean squared error of the lateral displacement estimates was reduced up to 55% when using the three-angle method. Since this compounding method only uses three acquisition angles, compound frame rates of approximately 40 Hz can be obtained, which makes the approach suitable for strain estimation in pulsating vessels.

The main goal of this study is to examine if the displacements resulting from this three-angle compounding also allow a better reconstruction of the elastic moduli than can be obtained through conventional single-angle imaging. To compare the accuracy of the reconstructions based on the two methods, simulated and experimental phantom data for various vessel geometries were generated. To our knowledge this is the first study that combines compounding methods for elasticity reconstructions in transverse cross sections of vessel shaped structures based on noninvasive ultrasound recordings obtained with a linear array transducer.

## 2. Methods

**Figure 1.**
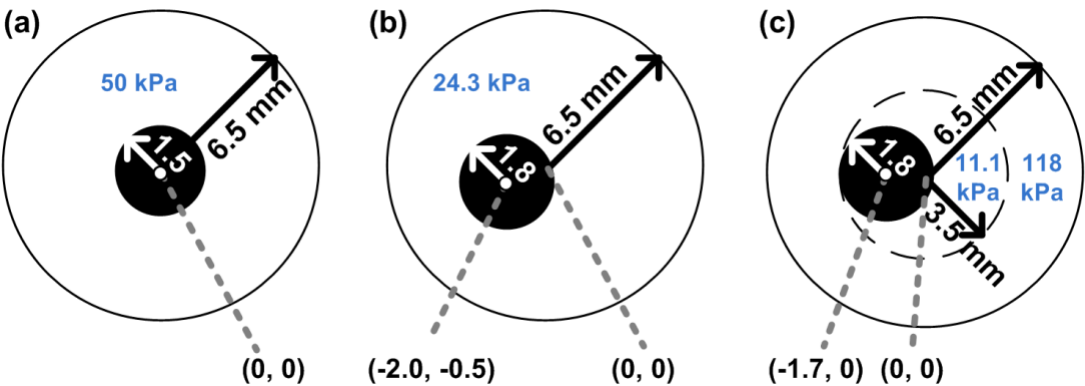
The geometries and Young's moduli of the vessels investigated. (**a**) A concentric homogeneous vessel; (**b**) An eccentric homogeneous vessel; (**c**) An eccentric vessel consisting of two layers with different stiffness.

To test our reconstruction methods, vessels with three different configurations were considered. The geometries are presented in Figure 1. The left and middle vessel are composed of homogeneous material with a concentric and an eccentric lumen, respectively. The right-most vessel resembles a vessel with a soft plaque. The latter vessel consists of two layers, a stiff outer layer and a softer inner layer. The Young's modulus of the inner layer is similar to that defined for soft necrotic core tissue in a previous theoretical study [16]. The Young's modulus of the outer layer is in the same order of magnitude as that reported for non-fibrotic tissue [9,27]. The modulus reconstruction was successively performed using: (1) 2D displacements derived from finite element solutions; (2) displacements estimated from simulated ultrasound data; and (3) 2D displacements estimated from experimentally obtained ultrasound data.

### 2.1. Modulus Reconstruction

To perform the estimation of the relative elasticity modulus, an iterative algorithm was used which requires the axial and lateral displacement fields of the tissue as input. The algorithm has been described extensively for radial displacement fields obtained from intravascular ultrasound (IVUS) data [33–35]. A brief explanation of its functioning for axial and lateral displacement field inputs will be provided next. The algorithm iteratively searches for an elastic modulus distribution which results in axial and lateral displacement fields [u_x_(μ) and u_y_(μ) ] that match the input displacement fields best ( $u_{x}^{m}$ and $u_{y}^{m}$) by minimizing a penalty function:

(1) $$\pi \left[\mu ,\mu_{0}\right]=\frac{1}{2}\pi_{u_{x}}+\frac{1}{2}\pi_{u_{y}}+\frac{\alpha }{2}\pi_{\mu }.$$

Here $\pi_{u_{x}}$ and $\pi_{u_{y}}$ are the displacement matching terms and π_μ_ is a smoothness term that restricts the amount of variation in the modulus field. α is a weighting factor that determines the amount the smoothness term contributes to the penalty function. α was set to 1e-9 for this entire study. *μ* is the shear modulus value, and *μ_0_* is a background shear modulus value with respect to which the smoothness function is normalized. *μ_0_* is similar to the function mean of the solution *μ*:

(2) $$\begin{array}{c} \mu_{0}=e^{\frac{\int_{\Omega }\ln\left(\mu \right)d\Omega }{\int_{\Omega }d\Omega }}\\ \pi_{u_{x}}=\int_{\Omega }{\left(u_{x}(\mu )-u_{x}^{m}\right)}^{2}d\Omega \\ \pi_{u_{y}}=\int_{\Omega }{\left(u_{y}(\mu )-u_{y}^{m}\right)}^{2}d\Omega \\ \pi_{\mu }=\int_{\Omega }\ln{\left(\mu /\mu_{0}\right)}^{2}d\Omega \end{array}$$

For the heterogeneous case with two layers, the constant *μ_0_* is divided into two regions, one for each layer, and is only held constant within each region. This is called a soft prior reconstruction. The separation of the normalizing function into two layers is required because a single normalizing constant for both regions would flatten out the contrast in modulus between the two layers, as demonstrated previously [35]. The relation between the shear modulus μ and the Young's modulus E is defined as follows:

(3) E=2μ(1+ν),

where *ν* is the Poisson ratio, which was assumed to be equal to 0.495 for all tissue layers. As a consequence the modulus images for the shear modulus and Young's modulus are the same except for a multiplication factor of ∼3. For the further analysis all modulus values were normalized by dividing the modulus values by the median of the modulus values obtained for the outer layer of each vessel. In this way the median of the modulus value for the outer vessel layer always equaled 1.

### 2.2. Mesh for Modulus Reconstruction

**Figure 2.**
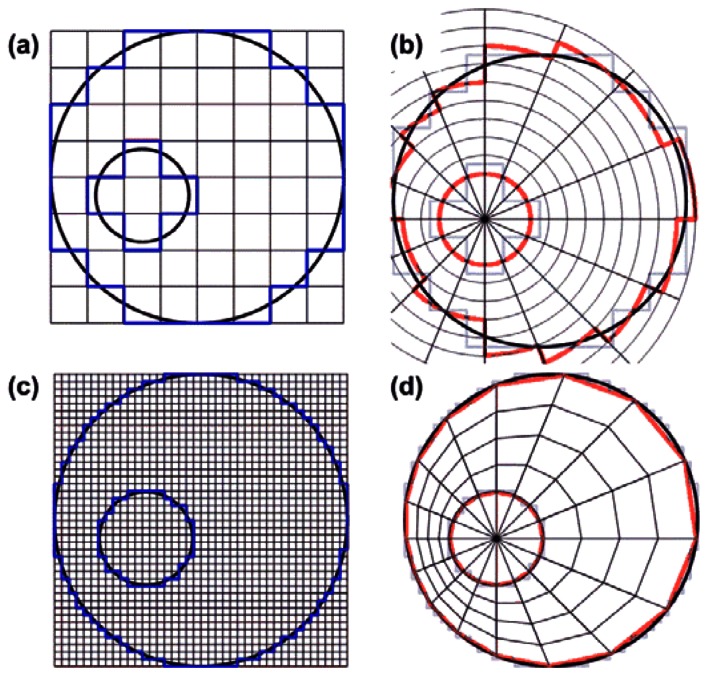
(**a**) Schematic drawing of the rectangular displacement grid as obtained from a linear array transducer; (**b**) Boundary discontinuities appear when projecting the data directly on a polar grid; (**c**) Upsampling and subsequent interpolation on the vascular finite element grid; (**d**) avoids boundary discontinuities to appear at the nodes.

To create the finite mesh for the modulus reconstruction, we segmented the inner and outer vessel boundaries, populated them with regularly spaced nodes and meshed them in a regular fashion, both radially and angularly, with respect to the lumen center Figure 2(d). We used this quasi-polar mesh to avoid element discontinuities at the vessel boundaries that would exist if we used a mesh that was rectangular as the grid obtained from the linear array ultrasound acquisitions Figure 2(a). In addition, the reconstruction algorithm applied the pressure as a surface traction, normal to the element edges on the inner lumen. This, to minimize shear artifacts that may exist at the inner lumen when applying a surface traction to elements on a regular Cartesian mesh. For computational simplicity, the measured displacements were interpolated onto the generated finite mesh such that the measured and predicted displacements existed at the same locations.

To obtain the finite mesh for a certain angle, the first displacement estimate within the vessel wall with respect to the lumen center, was considered as the inner radius value for that angle. Then, for the same angle the last displacement estimate within the vessel wall was determined and defined as the outer radius value for that angle. The procedure was repeated for each angle at an angular increment of 1° resulting in a value for the inner and the outer radius for each angle. Next, a small and a large ellipse were fitted through the inner and outer radius values using least squares fitting. Because the radii of the lumen ellipse were a little underestimated and the outer vessel radii were overestimated by the fitting procedure again discontinuities were generated at the boundaries. To reduce these discontinuities as well, the lumen ellipse was slowly expanded and the wall ellipse was slowly shrunk until all values laid within the ROI. Once the ellipses were known, the wall thickness for each angle was calculated (*D_wall_*), which was defined as the distance between the intersections of a line drawn at a certain angle from the lumen centre with the fitted inner and outer ellipses. For each angle the same number of displacement values were taken into account equidistantly divided over the distance between the inner and outer ellipse. The number of points at each angle was equal to:

(4) $$\#\text{points}=\frac{\underset{\theta }{\text{maxarg}}\;D_{\text{wall}}\left(\theta \right)}{2f_{p}c},$$

with c being the speed of sound (1,540 m/s), and f*_p_* the minimal sampling frequency on the polar grid, which was set to 7 MHz. Because a direct interpolation of the data from the rectangular grid onto the vascular mesh results in loss of points at the lumen-wall interface, the displacement data were first upsampled by a factor of 5 axially and laterally (Figure 2(a,b)). After that, the projection onto the IVUS grid was carried out using bilinear interpolation (Figure 2(d)).

### 2.3. Finite Element Modeling

As stated before, first the reconstruction algorithm was applied to axial and lateral displacement fields obtained by finite element modeling. Finite element models (FEMs) of the three vessel geometries were constructed using the Partial Differential Equation Toolbox of Matlab 2007a (MATLAB, The Mathworks, Natick, MA, USA). The displacement fields for the three different vessel configurations were calculated for an intra-luminal pressure increase of 4 mmHg under the assumption of plane strain. Over 60,000 two-dimensional linearly elastic finite elements were defined and distributed over the vessel volume. All elements were assumed to be nearly incompressible (Poisson's ratio, ν = 0.495) and isotropic. The Young's moduli of the various layers were set to the values shown in Figure 1 To prevent rigid body translation and to obtain unique FEM solutions, a highly compressible (ν = 0.001) and soft (E = 1 Pa) circular surrounding layer with a fixed outer boundary was temporarily added to the FEMs during the calculation of the axial and lateral displacement fields. The outer radius of this layer was 1 cm and its center corresponded to the point (0,0), see Figure 1. Because the FEM displacement fields were output on a triangular grid, and the linear array data is on a rectangular grid, the FEM displacements were interpolated on a rectangular ‘linear array’ grid using bilinear interpolation. The spacing of this grid was 158 μm axially and 135 μm laterally, which was equal to that obtained after displacement estimation using experimental ultrasound data.

### 2.4. Ultrasound Simulations

To test the strain estimation in combination with the reconstruction method in a controlled situation, ultrasound RF data were simulated using the ultrasound simulation software package Field II [36,37]. A linear array transducer was simulated with a center frequency (f_c_) of 8.7 MHz and 288 physical elements. The element pitch was 135 μm and the element height 6 mm. Each physical element was subdivided into 10 by 10 mathematical elements. RF data were simulated at a sampling rate of 117 MHz and down sampled to 39 MHz afterwards to match the experimental situation. The three times higher sampling rate during the simulations was chosen, because Field II uses digitally sampled versions of the excitation and impulse responses, which are continuous signals in reality. In the axial-elevational direction, a fixed focus of 2.5 cm was set and Hanning apodization was used in both transmit and receive to mimic an acoustical lens. In the axial-lateral direction, a fixed transmit focus of 2.5 mm was set. In transmit, no apodization was applied; in receive, dynamic focusing was applied with an F-number of 0.875. The maximum number of simultaneously active elements was restricted to 128. Lateral apodization with a Hamming window was applied in receive. Toward the edges of the transducer a lower, but symmetric, number of elements were active in receive and transmission. All these parameters were chosen to match the transducer used in the experiments. To generate the pre-deformation RF data, one million scatterers were randomly distributed over the cross sections of the vessels shown in Figure 1. The elevational thickness of the cross sections was assumed to be 1 cm. A plane strain condition was assumed. Therefore, the post-deformation position of each scatterer was defined as its pre-deformation position plus the axial and lateral displacement as obtained from the FEM. RF data of the vessel were simulated in pre- and post-deformation state for beam steering angles of –30°, 0° and +30°. Band-limited noise (3–11 MHz) was added to the simulated data to obtain a signal-to-noise ratio of 10 dB. To create the band-limited noise at the desired level, a region of interest (ROI) corresponding to the vessel wall area was selected in the zero degree acquisition. For this ROI the average energy per RF data sample point in the frequency band between 3 and 11 MHz was calculated. Simultaneously a white noise signal was generated that was filtered with a 3 to 11 MHz band-pass filter. For this filtered white noise signal the average energy per sample point was calculated also. The band-pass filtered white noise signal was then amplified and added to the noise-free simulated RF data such that the desired 10 dB signal-to-noise ratio was generated.

### 2.5. Phantom Experiments

In analogy with the finite element modeling and the simulations, vessel mimicking phantoms were constructed for each vessel geometry. The phantoms were constructed from various gelatin-agar solutions with Young's moduli as shown in Figure 1. The production of these phantoms as well as the estimation of the Young's moduli was described previously [38]. The phantoms were placed in a water tank and connected to a water column. A pressure step of 4 mmHg was applied to deform the tissue. RF data before and after the deformation were recorded for beam steering angles of –30°, 0°, and 30° using a Philips SONOS 7500 ultrasound system with an RF interface. A linear array transducer, L11-3 (f_c_ = 8.7 MHz, pitch = 135 μm, f_s_ = 39 MHz) was used to acquire the data. The focus was set at 2.5 mm.

### 2.6. Displacement Estimation

Before performing displacement estimation, all RF data were low-pass filtered to remove the signal caused by grating lobes. Additionally, a correction was applied to correct for the skewness of the beam-steered data. Both the grating lobe filtering and the correction for the skewness were described in detail previously [26]. A coarse-to-fine 2D cross-correlation based algorithm was used to obtain the displacement fields from the ultrasound data [39]. In four iterations the 2D motion was estimated in the tissue that occurred due to the intraluminal pressure increase. In each iteration the displacement of a certain point within the vessel wall was estimated by calculating the normalized cross-correlation function of a 2D kernel of RF data from the low pressure ultrasound recording (pre-deformation) within a larger search region of RF data from the high pressure acquisition (post-deformation). The 2D location of the peak of the resulting normalized cross-correlation function was regarded as the 2D tissue displacement due to the increase in intraluminal pressure. In the first iteration the pre-deformation kernel and post-deformation search region were both centered around the same point. In the remaining iterations, the post-deformation search region received an offset equal to the displacement value found in a preceding iteration to enhance the cross-correlation procedure. In between iterations one and two, and two and three, the sizes of the 2D kernels were decreased by a factor of 2 to improve displacement estimates by using more local information (the coarse-to-fine approach). The pre-deformation kernel size was 316 μm × 675 μm and the post-deformation search region size was 473 μm × 1,485 μm in the last two iterations. 2D parabolic interpolation of the cross-correlation function was applied to obtain displacement estimates at a sub-sample level [39,40]. These sub-sample displacement estimates were used to obtain a sub-sample shifted version of the post-deformation data. This shifted post-deformation data was input in the last iteration to enhance the performance of the cross-correlation peak finding procedure. The entire displacement estimation was repeated for every 158 μm axially and 135 μm laterally. In between iterations, displacements were filtered with a median filter with a kernel size of 9 times 9 displacement points. For the simulated data no filtering was applied after the final iteration. For the experimental data additional median filtering with a kernel size of 5 × 5 displacement points was applied after the final iteration for all displacement estimates that were detected to be outliers. Outliers were defined as displacement values that exceeded the median value of its 9 × 9 neighbors by more than 10 μm. To avoid losing displacement contrast at the lumen-vessel interface the kernel size of the median filter was reduced close to that interface.

Displacement estimation was carried out separately for each beam steering angle. The axial component was estimated directly from the non-steered 0° acquisitions both for the three-angle method and the conventional single-angle acquisition method. The lateral component was either estimated from the non-steered 0° acquisitions (single-angle imaging) or indirectly, by projecting the axial displacement estimates from the acquisitions at the positive and negative beam steering angles (three-angle compound imaging) using the equation:

(5) $$u_{\text{lat},0}=\frac{u_{ax,\theta }-u_{ax,-\theta }}{2\sin\theta },$$

where u_ax,θ_ and u_ax,-θ_ are the axial displacements estimated at the positive and negative beam steering angle, respectively. u_lat,0_ is the lateral displacement component.

### 2.7. Analysis

To determine the performance of the modulus reconstruction, the median and interquartile range (IQR) of the absolute differences between the axial and lateral displacement input and output of the reconstruction method were calculated. For example, for the axial component this absolute difference Δu_x_ was calculated following:

(6) $$\Delta u_{x}(r,\theta )=|u_{x}(r,\theta )-u_{x}^{m}(r,\theta )|,$$

where u_x_ is the reconstructed axial displacement, and u_x_^m^ is the estimated axial displacement for a point on the mesh with radius r, and angle θ.

Next to these absolute displacement differences, the median value and the IQR of the relative modulus estimates over the vessel wall were calculated. For the heterogeneous vessel, the modulus analysis was performed separately for both layers.

## 3. Results and Discussion

### 3.1. Results

#### 3.1.1. Finite Element Modeling

**Figure 3.**
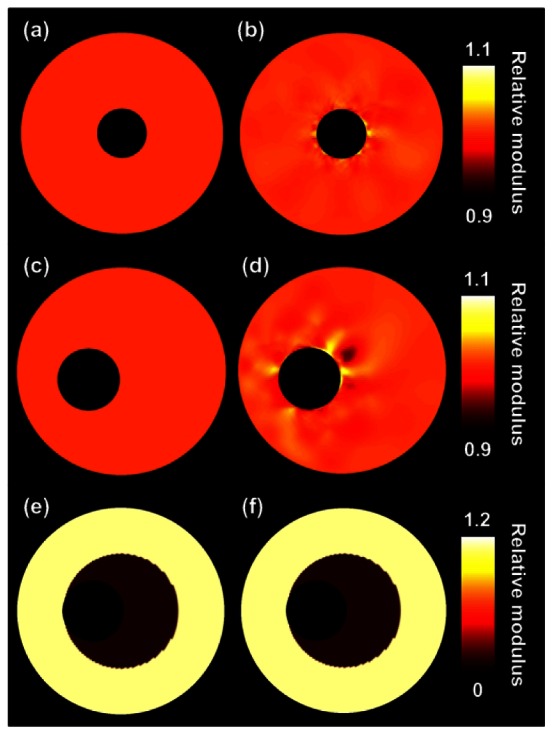
(**a**), (**c**) and (**e**) are the true relative modulus images for the three investigated vessel phantoms. (**b**), (**d**) and (**f**) are the corresponding relative modulus images derived from the displacement fields obtained directly from the finite element models.

Figure 3 shows the true relative modulus images and the relative modulus images based on the FEM displacement fields for all three vessel configurations. Thus, no imaging was involved for the generation of these modulus images. As can be observed, in general theory and finite element image match well, although some errors appear in the vicinity of the lumens. These errors seem larger for the homogeneous eccentric vessel than for the homogeneous concentric vessel.

The left column of Table 1 presents the corresponding median modulus and IQR values for the FEM images. For the heterogeneous vessel a differentiation is made between the soft inner layer and the stiff outer layer. The errors for the homogeneous cases are small, interquartile moduli range from 0.997 to 1.003. For the homogeneous eccentric case these errors are slightly larger and range from 0.995 to 1.006. For the heterogeneous case theoretically moduli of 0.094 and 1 ought to be detected for the inner and outer layers, respectively. For the inner layer a median modulus and an IQR of 0.105 (0.105, 0.105) is obtained. For the outer layer an IQR of 0.996–1.006 is found, thus the contrast is slightly reduced.

#### 3.1.3. Ultrasound Simulations

**Figure 4.**
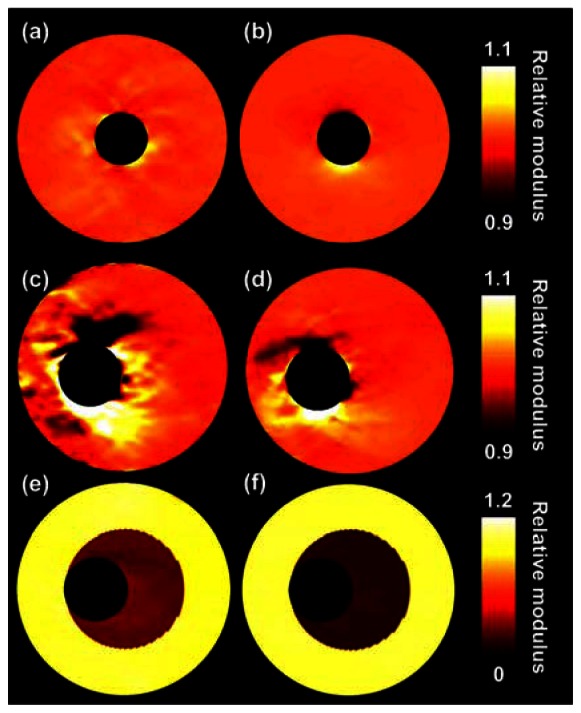
Relative modulus images for the three vessels obtained from ultrasound simulations. (**a**), (**c**) and (**e**) are estimated with conventional single-angle imaging; (**b**), (**d**) and (**f**) are based on three-angle compounded displacement fields.

Figure 4 presents the reconstructed modulus images obtained for the ultrasound simulations. The left and right columns show the results without and with compounding, respectively. The quality of the modulus reconstruction improves visually for all three configurations when using compounding. In the two homogeneous cases, the errors close to the lumen-vessel interface reduce and in the heterogeneous case, an increase in contrast between the soft and the stiff layer is observed. As can be observed in Table 1 the IQR of the reconstructed modulus is decreased by at least a factor of 2 when performing compounding. As expected, because compounding aims at improving the lateral displacement component, especially in that direction the displacements are matched more successfully. The absolute difference between input and output lateral displacements is reduced by a factor of 2 to 3 with compounding. In axial direction displacements are also better matched, although the improvements are smaller. The contrast between the two layers of the heterogeneous phantom is also closer to the true contrast value when performing compounding.

#### 3.1.4. Phantom Experiments

**Figure 5.**
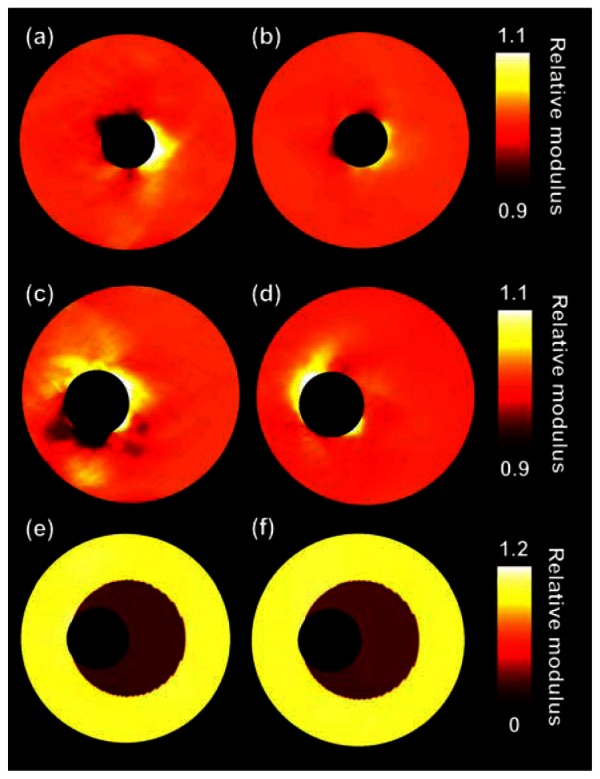
Relative modulus images for the three vessels obtained from phantom experiments. (**a**), (**c**) and (**e**) are estimated with conventional single-angle ultrasound imaging. (**b**), (**d**) and (**f**) are based on three-angle compounded displacement fields.

Figure 5 presents the relative modulus images that are reconstructed for the experimental phantom data. Table 2 provides the corresponding quantitative values. Again, a visual as well as a quantitative improvement in reconstruction accuracy method is observed for the homogeneous cases when applying three-angle compounding. The lateral displacements are again a factor of 2 to 3 better matched. Also, the IQR of the modulus reduces two-fold with respect to the single-angle acquisition. For the two layers of the heterogeneous vessel again a better match is found in the lateral direction (factor 1.8), although the modulus IQR for the inner layer is similar with and without compounding. For the outer layer only a small reduction in the modulus IQR is observed when performing compounding. Opposed to the simulation results, the modulus contrast between the two layers decreased when using compounding in this experiment.

### 3.2. Discussion

The results illustrate that the combination of the reconstruction algorithm, the interpolation method, and the iterative 2D cross-correlation based displacement estimation techniques allow an accurate reconstruction of relative Young's moduli based on linear array ultrasound data. As can be expected, because the lateral displacements improve with compounding [17], the lateral displacements input to the reconstruction algorithm were a better match with the displacements output by the reconstruction algorithm when using three-angle compounding instead of single-angle imaging. In general, also a better match was found in axial direction, although the improvements were less pronounced in that direction. The better match in the axial direction was probably due to the coupling of the axial and lateral data in the reconstruction algorithm. The improved match in both directions also allowed more accurate modulus reconstructions in almost all cases: the variation in relative modulus for the homogeneous regions was smaller, as illustrated by the decrease in the modulus IQR. The only case that did not show a clear improvement in modulus IQR was the experiment with the heterogeneous phantom, although the modulus IQR did slightly reduce for the outer layer with compounding. Opposite to the results for the simulations, we observed an increase in the contrast between the layers with compounding. However, it should be noted that the true modulus contrast was not perfectly known in the experiments. The used modulus values were determined using pressurization experiments which only results in a rough indication of the real moduli [38]. As a consequence, the true modulus value for the inner layer might have been slightly underestimated. In addition, the modulus value for the outer layer might have been slightly overestimated. Both the underestimation and the overestimation would lead to a smaller modulus contrast in modulus. Nevertheless, overall we observe an improvement in reconstruction accuracy, although the improvement in the modulus reconstruction is less convincing than the improvement in the lateral displacement component.

As aforementioned, most errors in the reconstructed images were observed at the lumen-vessel interface. These errors were observed even in the FEM results (Figure 3), where no imaging was included yet. Thus, the errors are not solely caused by imperfections in the displacement estimation. To check whether the errors were coming from the modulus reconstruction we did some additional experiment, which is not reported in this manuscript, in which the analytically solved displacement field of the homogeneous concentric vessel was input to the modulus reconstruction algorithm. The resulting modulus distribution was completely homogeneous. This automatically implies that the interpolation steps are causing the errors in the vicinity of the lumen: step 1 from the triangular finite element grid to the ‘linear array transducer’ grid and step 2 from the ‘linear array transducer’ grid to the polar grid. Since the spacing of the polar grid is most dense close to the lumen-wall interface, and the rectangular grid is equidistant everywhere, increased errors are observed in the vicinity of the lumen-wall interface. For the eccentric cases these errors are larger, because the grid is more irregular.

In previous modulus reconstruction studies using intravascular ultrasound data, no specific increase of error in the vicinity of the lumen interface was observed. This can probably be explained by the fact that IVUS imaging is already performed in a polar grid, thus no interpolation is required. The errors are in the order of 10% when performing compounding and larger without compounding, which seems quite large. However, also without compounding the errors are hardly noticeable when considering a contrast difference between different parts of a plaque of a factor of 10 as is illustrated for instance by Figure 5(e,f). Contrast differences between lipids and other plaque components in the order of a factor 10 have been reported before [9,10,16]. Of course the investigated vessel mimicking phantoms are not as complex structures as real carotid arteries, therefore it is advised to repeat this study *in vitro* or *in vivo* for real carotid arteries to investigate the performance of the algorithms in those complex situations. The studied phantoms did for instance not contain a thin fibrous cap surrounding the soft plaque region, which might also influence the performance of the displacement estimation and the modulus reconstruction. Furthermore, the wall thickness of the phantoms is up to 6.5 mm which is much larger than the wall thickness of carotid arteries in the early stages of atherosclerosis which is generally less than 2 mm. The phantoms used are more representative for the severely stenotic wall. Of course the developed methods might be applied to examine the lipid content in those severe cases, although early prevention is of course more desired. Fortunately, several studies have already demonstrated noninvasive strain imaging in thin walled carotid arteries, which suggests that modulus reconstruction should also be possible in such thin walls [21,41,42].

Another point of discussion is the fact that we reconstructed relative modulus images instead of absolute modulus images. The reason we did not investigate absolute moduli, is because that also requires knowledge of the frame-to-frame intraluminal hydrostatic pressure change. Although the hydrostatic pressures were known in the present study, these pressures will not be known in an *in vivo* setting, because it is impossible to obtain a very accurate noninvasive measurement of the instantaneous blood pressure in the carotid artery.

**Figure 6.**
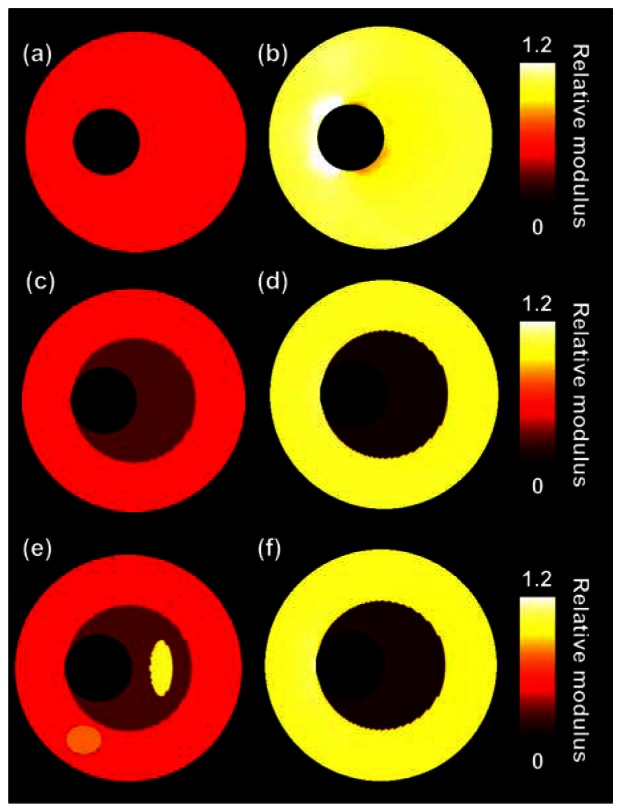
Modulus reconstructions for the heterogeneous two-layered phantom for different soft-prior settings. On the left the regions are shown that were considered to have a different modulus. On the right the corresponding relative modulus reconstruction using soft priors.

Finally a short discussion about the applicability of the soft prior condition *in vivo*. Figure 6 shows three different modulus reconstructions for the heterogeneous phantom. The top row shows the results without soft prior assumption. The center and bottom row show results with soft priors after defining two and four regions, respectively. The selected soft prior regions are indicated with different colors in the column on the left. As can be observed the soft prior assumption is necessary for a correct detection of the contrast between the different layers, as also illustrated before [35]. Without soft prior the contrast between the two layers disappears completely. The definition of more regions than there actually are in reality does not have much impact on the reconstruction. For *in vivo* application of the soft prior method we suggest defining regions based on echolevel. In case a region cannot be nicely delineated on an echo image, then the region should be subdivided into smaller regions, because, as aforementioned, the subdivision into some additional soft prior regions does not seem to lead to a deviation of the reconstructed modulus. Of course it can be expected that regions cannot be subdivided infinitely, because at a certain point it will no longer be possible to get accurate modulus values. This should be investigated in more detail in an additional study. Furthermore, it should be investigated what the impact on the modulus reconstruction performance is when the soft prior regions are not delineated correctly.

## 4. Conclusions and Future Work

Accurate modulus reconstructions for transverse cross sections of vascular structures can be obtained given axial and lateral displacement estimates which are estimated based on raw radio frequency ultrasound data obtained noninvasively with a linear array transducer. Displacements obtained using a recently developed three-angle compounding method allow a more accurate reconstruction of the modulus than can be obtained from displacements estimated from conventional single-angle images. The next step will be to explore the limits of the proposed modulus reconstruction method and to determine its performance *in vitro* and *in vivo*.

## Figures and Tables

**Table 1.** Median and interquartile ranges (IQR) of the modulus reconstruction of the three vessels based on finite element modeling (FEM) and simulations. The median and IQR of the absolute differences between the input and output displacement fields are reported also.

**Vessel**	**FEM**	**Simulations**		
	**Relative Modulus**	**Relative Modulus**	**Ax diff (μm)**	**Lat diff (μm)**
	**Median (IQR)**	**Median (IQR)**	**Median (IQR)**	**Median (IQR)**
(a)°	1.000 (0.997–1.003)	1.000 (0.996–1.005)	0.7 (0.3–1.3)	4.8 (2.3–8.3)
(a)^C^		1.000 (0.997–1.002)	0.5 (0.2–0.8)	1.3 (0.6–2.4)
(b)°	1.000 (0.995–1.006)	1.000 (0.982–1.030)	1.5 (0.7–2.8)	8.0 (3.9–13.6)
(b)^C^		1.000 (0.991–1.015)	1.3 (0.6–2.3)	3.1 (1.4–5.7)
(c)_in_°	0.105 (0.105–0.105)	0.236 (0.228–0.252)	2.4 (1.0–4.4) *	8.3 (3.8–14.0) *
(c)_in_^C^		0.128 (0.128–0.128)	1.2 (0.5–2.0) *	3.4 (1.6–5.9) *
(c)_out_°	1.000 (0.996–1.006)	1.000 (0.984–1.015)	2.4 (1.0–4.4) *	8.3 (3.8–14.0) *
(c)_out_^C^		1.000 (0.993–1.000)	1.2 (0.5–2.0) *	3.4 (1.6–5.9) *

(a) = concentric homogeneous vessel; (b) = eccentric homogeneous vessel; (c)_in_ = inner layer heterogeneous vessel; (c)_out_ = outer layer heterogeneous vessel,

° = single-angle,

C = with compounding.

* = No differentiation between the two layers of the heterogeneous vessel.

**Table 2.** Median and interquartile ranges (IQR) for the modulus reconstruction of the three vessels based on experiments. The median and IQR for the absolute differences between the input and output displacement fields are reported also.

**Vessel**	**Experiments**		
	**Relative Modulus**	**Ax diff (μm)**	**Lat diff (μm)**
	**Median (IQR)**	**Median (IQR)**	**Median (IQR)**
(a)°	1.000 (0.994–1.004)	1.0 (0.5–1.9)	3.4 (1.6–5.7)
(a)^C^	1.000 (0.997–1.002)	0.8 (0.3–1.5)	1.0 (0.5–1.7)
(b)°	1.000 (0.988–1.015)	1.5 (0.4–2.6)	4.1 (2.0–7.0)
(b)^C^	1.000 (0.996–1.008)	1.3 (0.5–2.1)	1.6 (0.8–2.8)
(c)_in_°	0.200 (0.195–0.203)	3.1 (1.5–5.3) *	3.6 (1.7–6.1) *
(c)_in_^C^	0.214 (0.210–0.220)	2.6 (1.2–5.5) *	3.4 (1.0–3.5) *
(c)_out_°	1.000 (0.992–1.009)	3.1 (1.5–5.3) *	8.3 (1.7–6.1) *
(c)_out_^C^	1.000 (0.996–1.008)	2.6 (1.2–5.5) *	3.4 (1.0–3.5) *

(a) = concentric homogeneous vessel; (b) = eccentric homogeneous vessel; (c)_in_ = inner layer heterogeneous vessel; (c)_out_ = outer layer heterogeneous vessel,

° = single-angle,

C = with compounding.

* = No differentiation between the two layers of the heterogeneous vessel.
